# The impact of gender and sex on diagnosis, treatment outcomes and health-related quality of life in patients with axial spondyloarthritis

**DOI:** 10.1007/s10067-022-06228-6

**Published:** 2022-06-28

**Authors:** Helena Marzo-Ortega, Victoria Navarro-Compán, Servet Akar, Uta Kiltz, Zoë Clark, Elena Nikiphorou

**Affiliations:** 1 NIHR Leeds Biomedical Research Centre, Leeds Teaching Hospitals Trust and Leeds Institute of Rheumatic and Musculoskeletal Medicine, University of Leeds, Leeds, UK; 2 University Hospital La Paz, IdiPaz, Madrid, Spain; 3 Department of Internal Medicine, Division of Rheumatology, Katip Çelebi University, İzmir, Turkey; 4 Rheumazentrum Ruhrgebiet, Herne, and Ruhr-Universität Bochum, Bochum, Germany; 5 Patient Author, Norwich, UK; 6 Centre for Rheumatic Diseases, King’s College London, London, UK; 7 Rheumatology Department, King’s College Hospital, London, UK

**Keywords:** Axial spondyloarthritis, axSpA, Females, Gender, Quality of life, Treatment response

## Abstract

Axial spondyloarthritis (axSpA) is a chronic inflammatory rheumatic condition, historically considered a predominantly male disease. However, increasing evidence suggests a more equal prevalence between men and women. Of the limited research conducted to date, it is apparent that gender differences exist in terms of time to diagnosis, treatment outcomes and health-related quality of life (HRQoL). Despite this, women are underrepresented in clinical trials and most studies do not stratify by gender to identify potential differences in terms of disease manifestations and treatment response. In this perspectives article, we reflect on the potential biological and social factors contributing to these differences and propose three key areas of education and research that should be prioritised in order to address the unmet needs of female patients with axSpA, namely: (1) to identify ways to increase awareness of disease occurrence in female patients among healthcare professionals (HCPs), (2) to improve understanding of gender differences in disease manifestation and outcomes, and (3) to conduct gender-stratified clinical trials with a representative sample of female patients.

**Supplementary Information:**

The online version contains supplementary material available at 10.1007/s10067-022-06228-6.

## Introduction

Axial spondyloarthritis (axSpA) affects the spine and sacroiliac joints and is characterised by inflammatory back pain, peripheral musculoskeletal and extra-musculoskeletal manifestations, with significant impact on physical functioning and ability [1–3]. AxSpA encompasses a broad spectrum of disease including non-radiographic (nr-axSpA) and radiographic axSpA (r-axSpA), also known as ankylosing spondylitis (AS) [4]. Current disease management focuses on pharmacological interventions, including non-steroidal anti-inflammatory drugs (NSAIDs) as a first-line treatment option and advanced therapies including biologics (such as tumour necrosis factor [TNF] and interleukin [IL]-17 inhibitors) and other disease modifying anti-rheumatic drugs (DMARDs). Non-pharmacological interventions, such as exercise and physiotherapy, are also recommended in order to reduce pain and conserve function and mobility [5–7].

Historically, axSpA was considered to be a predominantly male disease, with early studies estimating a tenfold higher prevalence in men vs women [8]. This gender disparity has declined over time due to the increased availability and understanding of diagnostic tools, with recent data suggesting a prevalence ratio for r-axSpA in the range of 1.2 to 2:1 in men vs women [9–11]. Nr-axSpA is generally considered to have a more equal distribution between genders [10].

Despite the increased availability of different treatment options [12] and improved recognition of axSpA in females [9–11], the limited evidence to date has highlighted inequalities between male and female patients with axSpA [13–15]. This perspectives article was conceived and developed by a working party comprising of a group of expert rheumatologists and a patient living with axSpA. Here, we reflect on relevant published literature (identified during a pragmatic review of the literature; see supplementary materials for details) and discuss gender differences^1^ in terms of time to diagnosis, treatment outcomes and health-related quality of life (HRQoL). We also consider the potential biological and social factors underpinning such differences and propose key areas of education and research that should be prioritised to address the needs of female patients with axSpA.

## Diagnostic delay in female patients with axSpA

Compared to other rheumatic diseases, axSpA is known to have substantially longer diagnostic delay which averages approximately 7 years compared to 3 years for psoriatic arthritis (PsA) and 2 years for rheumatoid arthritis (RA) [16]. Strikingly, female patients with axSpA experience longer diagnostic delays than their male counterparts (8.2 years vs 6.1 years) and have a higher number of visits to general practitioners (82.1% vs 74.7%), osteopaths (24.4% vs 13.3%) and physiotherapists (49.5% vs 34.5%) before being diagnosed [13]. These findings are consistent with reports from an early disease detection cohort that male patients with axSpA are younger at the time of diagnosis (27.4 ± 7.5 years in males vs 29.5 ± 7.8 years in females) [17].

In addition, men are more likely to receive a correct first diagnosis of axSpA compared with women, with 30% of men receiving a first correct diagnosis compared with just 11% of women [18]. Many factors are thought to contribute to this longer diagnostic delay, including inadequate healthcare professional (HCP) knowledge [19], historical biases and poor communication between HCPs [20], resulting in a lack of awareness of potential gender differences in disease manifestation, leading to misdiagnoses, mainly of fibromyalgia [21, 22]. Common myths continue to exist surrounding axSpA, meaning that many HCPs still view axSpA as a male disease and, despite advances in imaging technology, difficulties and inconsistencies remain regarding the use of magnetic resonance imaging (MRI) for axSpA, further impacting the diagnostic delay in females [21].

Diagnostic delay can contribute towards the burden of disease on both a patient and society level [23] and impacts functional ability. Longer time to diagnosis has been associated with worse Bath Ankylosing Spondylitis Functional Index (BASFI) and Bath Ankylosing Spondylitis Metrology Index (BASMI) scores, reduced spinal mobility and greater radiographic progression [23]. However, it is unclear whether these data are applicable to both sexes since these reports derivate from cohorts comprising primarily of men, so more data are required to understand whether a longer diagnostic delay in women leads to increased radiographic progression. Furthermore, diagnostic delay has a significant impact on HRQoL, with studies reporting worse Ankylosing Spondyloarthritis QoL (ASQoL) questionnaire scores (indicating greater impairments to HRQoL) in people experiencing longer delays to diagnosis [24, 25]. Findings from qualitative research emphasise this further, by highlighting that the meandering and frustrating diagnostic journeys women experience can contribute to substantial psychological distress and significant suffering which could be prevented with earlier diagnosis and appropriate intervention [26, 27]. Aside from the impact on the individual, delayed diagnosis and treatment naturally come with wider, societal impact [23]. As a result, the treatment costs associated with diagnostic delays and unnecessary healthcare utilisation resulting from increased frequency of visits to general practitioners and specialist services, unnecessary surgeries and inappropriate treatments are enormous [28, 29].

## Gender differences in treatment use and outcomes

Women are often underrepresented in clinical research and are less likely to participate due to factors such as contraceptive restrictions, resulting in the majority of patients enrolled in randomised controlled trials being male [14, 30, 31]. In addition, female patients with axSpA have a lower probability of achieving remission compared to males [32]. To date, there are no published data for randomised controlled trials specifically designed to examine gender differences in treatment response in patients with axSpA, with most available evidence based on observational studies or post hoc analyses from clinical trials [15, 33–36]. Consequently, the current understanding of how different underlying factors (biological or social) influence treatment outcomes in male and female patients with axSpA is limited.

To explore gender differences in treatment response, van der Horst-Bruinsma and co-authors pooled data stratified by gender from four interventional or observational trials, all examining the efficacy and safety of TNF inhibitors in patients with AS [15]. Mean baseline data indicated that women had a higher age at disease onset, shorter disease duration and lower levels of C-reactive protein (CRP)—a key indicator of inflammation and predictor of clinical response to TNF inhibitors [37]. Women had significantly lower improvements in week 12 efficacy outcomes (including Ankylosing Spondylitis Disease Activity Score [ASDAS], Bath Ankylosing Spondylitis Disease Activity Index [BASDAI] and BASFI) relative to men, despite having a later onset of disease [15]. However, as highlighted by the authors, the original studies were not designed to evaluate these differences between male and female participants, and hence do not account for any potential biases, including those which may have been introduced by HCPs regarding axSpA and gender. Furthermore, the original studies do not account for potential differences in MRI-detected bone marrow oedema, and only partially for differences in CRP levels. This is relevant since objective evidence of inflammation as reflected on MRI and CRP are the main predictors for treatment response to biologic DMARDs in AS, and it is unclear whether any difference in the levels of inflammation may be found between males and females at baseline.

Such research indicates differences in treatment response between men and women, with more limited improvements observed in women, and highlights the need for more research to explore this further. Indeed, only a few studies reporting results from clinical trials that stratify treatment response by gender in patients with axSpA have been published in the last years [33–36]. Dougados et al. examined treatment response in terms of improvements in tenderness at entheseal sites using the Maastricht Ankylosing Spondylitis Enthesitis Score (MASES) [38] in patients with axSpA treated with certolizumab pegol for up to 4 years [33]. Female patients (*n* = 83) reported higher baseline MASES scores (indicating worse tenderness at entheseal sites) than male patients (*n* = 135), and although greater improvements in MASES were reported for female patients over the 204-week study period, week 204 MASES scores were still higher in females patients compared to male patients [33]. Landewé et al. examined the influence of gender, age and axSpA subpopulations on clinical remission following dose reduction of certolizumab pegol during the open-label induction period of a phase 3b study. The authors concluded that a reduced maintenance dose was suitable for patients who achieved sustained remission following 1 year of treatment, regardless of gender. However, only a third of participants in the trial were female, of whom a third (66/222 female patients) achieved sustained remission within the 48-week induction period therefore entering the 48-week maintenance period, compared with nearly 50% (247/514) of male participants [34, 39].

Beyond TNF inhibitors, studies on the efficacy of drugs with other modes of action report higher relative responses in men compared to women. Braun et al. investigated the efficacy of secukinumab in patients with nr-axSpA grouped by disease activity as assessed by CRP levels, MRI scores, human leukocyte antigen HLA-(B27) status and sex [35]. Authors reported higher relative responses to secukinumab in male participants (51.2% of males achieved Assessment of SpondyloArthritis international Society 40% [ASAS40] vs 31.7% for females) [35]. Furthermore, the ASAS40 response seen in females (31.7%) was comparable to the placebo response in males (30.8%) and only slightly higher than the placebo response seen in females (25.3%). Thus, while a significant difference in treatment response was detected in males, this was not seen in the female participants [35]. Interestingly, the same study also demonstrated a greater severity of sacroiliac joint (SIJ) oedema on MRI in males (mean baseline SIJ MRI score ≥ 2 was 53% in males vs 44% in females), with higher scores corresponding to better treatment response [35]. Similarly, another post hoc analysis investigating the efficacy of secukinumab conducted by Magrey et al. found that female patients had a delayed response to treatment compared to males (37.5% of female patients achieved ASAS40 by week 16 compared with 46.3% of male patients) [36]. Taken together, these data suggest that the challenge at the clinical level would be to identify the subset of females who are more likely to have higher inflammation levels on MRI.

Observational studies also suggest differences in terms of treatment adherence and drug use in female patients with axSpA. Cohort studies such as that reported by Rusman et al. suggest that women have significantly shorter treatment periods compared with men (33.4 vs 44.9 months, respectively) and are more likely to switch between biologic treatments (26.9% switching vs 16.3%) [14], with lack of efficacy being the most commonly reported reason for stopping or switching treatment [40, 41]. Use of biologics is typically higher in men vs women, and women are more likely to be treated with intra-articular steroids, aminosalicylates and corticosteroids [42, 43]. However, it is unclear why these differences arise. The general lack of evidence regarding gender/sex differences in clinical trials for patients with axSpA contributes to a limited awareness of differences in disease manifestation and knowledge surrounding the efficacy of treatments, specifically for women.

## Gender differences in HRQoL outcomes

The clinical manifestations of axSpA that lead to reduced physical functioning and restricted participation in daily activities can result in impaired HRQoL and reduced life-satisfaction [44]. Female gender has been disproportionately associated with impaired HRQoL in patients with axSpA, as reported using multiple tools such as the Short-Form (SF-36) Health Survey mental component score, ASQoL and the ASAS Health Index (ASAS HI) [45–47]. Many factors have been explored which could explain the impaired HRQoL reported in women, including fatigue, pain, sleep disturbances and increased disease activity, all of which have been reported to affect women more than men [48, 49]. It has been hypothesised that fatigue, widespread pain and sleep disturbances can often lead to misdiagnoses of fibromyalgia in females with axSpA [50].

To better understand and address the gender differences in HRQoL among patients with axSpA, further research is needed into why women experience greater central pain and how pain and other overlapping clinical features of fibromyalgia can be differentiated from axSpA. Reasons for greater workplace disability should also be explored to disentangle factors that are associated with underlying health conditions (either directly or indirectly) and potential unrelated factors such as culture and education.

## Biological differences between male and female patients with axSpA

An important genetic predisposition in axSpA is the association with the HLA-B27 allele. HLA-B27 carriership has been found to be more prevalent in men vs women [13], which could contribute towards the differing presentation of axSpA between sexes, such as radiographic progression [22]. HLA-B27 is also associated with a greater chance of axSpA detection by MRI [51] and better treatment response [22]. Biological sex differences in patients with axSpA have also been identified in terms of gene expression. Gracey et al. found that 291 immune modulator genes were uniquely expressed in female patients vs 1522 genes in males, and found higher levels of cells positive for the inflammatory markers IL-23 and IL-17A in affected joints of men vs women [52]. These findings suggest distinct differences in the immunological profile of men vs women with axSpA, with higher inflammatory cellular markers in men potentially influencing response and adherence to treatments. However, it is important to emphasise that differences in HLA-B27 positivity could be due to a higher rate of misclassification in women, particularly in nr-axSpA. Furthermore, Rusman et al. suggest that sex hormones may play a role in disease manifestations, highlighting the anti-inflammatory effect of oestrogen on SpA manifestations by inhibiting TNF alpha, and the precursor to testosterone’s influence on the onset and severity of AS [14]. Further evidence is needed to address whether different hormonal milieus between sexes account for any differences observed between men and women.

Biological sex differences in body mass index (BMI) in patients with axSpA have also been examined and associated with disease activity and treatment response. Studies have reported a higher BMI in males than females with axSpA [48, 49]. However, female patients with axSpA have been reported to have a higher fat mass index (FMI) and more likely to be obese than their male counterparts (28.6% compared to 7.1%), which has been linked to higher disease activity [53]. Furthermore, there is evidence to suggest that women with axSpA are less likely to engage in physical exercise [13], which could contribute to the higher observed FMI and lead to impairments in HRQoL [54].

These biological differences may likely influence the diverse clinical manifestation of axSpA between sexes, with women presenting mainly with fatigue, stiffness, enthesitis, widespread pain and peripheral disease, and men presenting with more structural damage on radiography and higher inflammatory markers [48, 52, 55]. The differences in clinical manifestation could contribute to difficulties in diagnosis and suboptimal treatment strategies. A study by Ortolan et al. analysing gender differences at the time of axSpA diagnosis reported a higher incidence of HLA-B27 and imaging positivity in males compared with females (80% vs 60% for HLA-B27; 78% vs 64% for MRI or radiographic imaging), but concluded that both factors still play an important role in the diagnosis of females with axSpA [17]. It has also been reported that pain perception and coping strategies differ between women and men as a consequence of bio-psychosocial influences such as hormones, endogenous opioid functions and genetic factors [46]. In male patients with axSpA, disease activity, specifically if measured using ASDAS, is significantly associated with inflammatory lesions on SIJs on MRI, but not in female patients [56, 57]. This could mean that the assessment of disease activity using tools such as BASDAI and ASDAS may not completely capture disease activity in women. Another consideration, however, is the potential risk of overdiagnosis if applying the ASAS classification criteria in the clinical setting. Indeed, according to the so-called clinical arm of the ASAS criteria [4], classification as nr-axSpA can occur in the virtual absence of imaging inflammation or an abnormal CRP, hence leading to potential misclassification of patients, often female, with other conditions such as fibromyalgia.

## Social differences between male and female patients with axSpA

Results from an analysis of gender differences in the patient journey to diagnosis from the European Map of Axial Spondyloarthritis (EMAS) found that women with axSpA were more likely to have a university-level qualification [13]. Despite this, women with axSpA are more likely to be homemakers or on temporary sick leave, and less likely to work full-time compared with men [13, 49]. In a cross-sectional study examining work and family life in patients with axSpA, patients were approximately 50% more likely than the general population to have never been married and 30% more likely to be divorced, with more women reporting being divorced than men [58]. When compared with the general population, a smaller proportion of women in the cross-sectional study had children (54.7% observed vs 64.9% expected) in comparison to men (54.5% observed vs 54.2% expected) [58]. This suggests that women may be concerned about how their condition or medication use could influence pregnancy, and how their condition may affect their ability to care for young children. In addition, female gender is associated with sexual activity problems as a consequence of the pain, stiffness and low moods patients experience, resulting in impaired HRQoL and decreased functionality [59, 60]. However, no study has specifically examined the association between these social factors and their influence on treatment efficacy, response or adherence.

Targeted social support for working-aged women with axSpA and improved knowledge surrounding medication use and pregnancy outcomes are necessary to address the complex social factors underpinning gender differences in patients with axSpA. Importantly, axSpA is associated with work disability [61] with women having a risk of work disability more than three times that seen in the general population (14.8% work disabled compared to 4.6% expected), and greater than that observed in men with axSpA (12.7% work disabled) [58]. This can have a significant financial and humanistic burden, with evidence suggesting that work productivity loss represents between 10 and 17% of annual costs for patients with axSpA [61].

## Discussion

Current evidence suggests that sex/gender differences exist in terms of time to diagnosis [13, 17], treatment outcomes [15, 33, 35, 36] and HRQoL [45–47] in patients with axSpA. These differences may be related to biological factors such as differing genetic and immunological profiles [13, 22, 52], or social factors such as employment and marital status [13, 49, 58]. However, due to a lack of research examining biological sex differences and poor female representation in clinical trials generally, factors contributing to poorer outcomes in women with axSpA are still largely unknown.

**Fig. 1:**
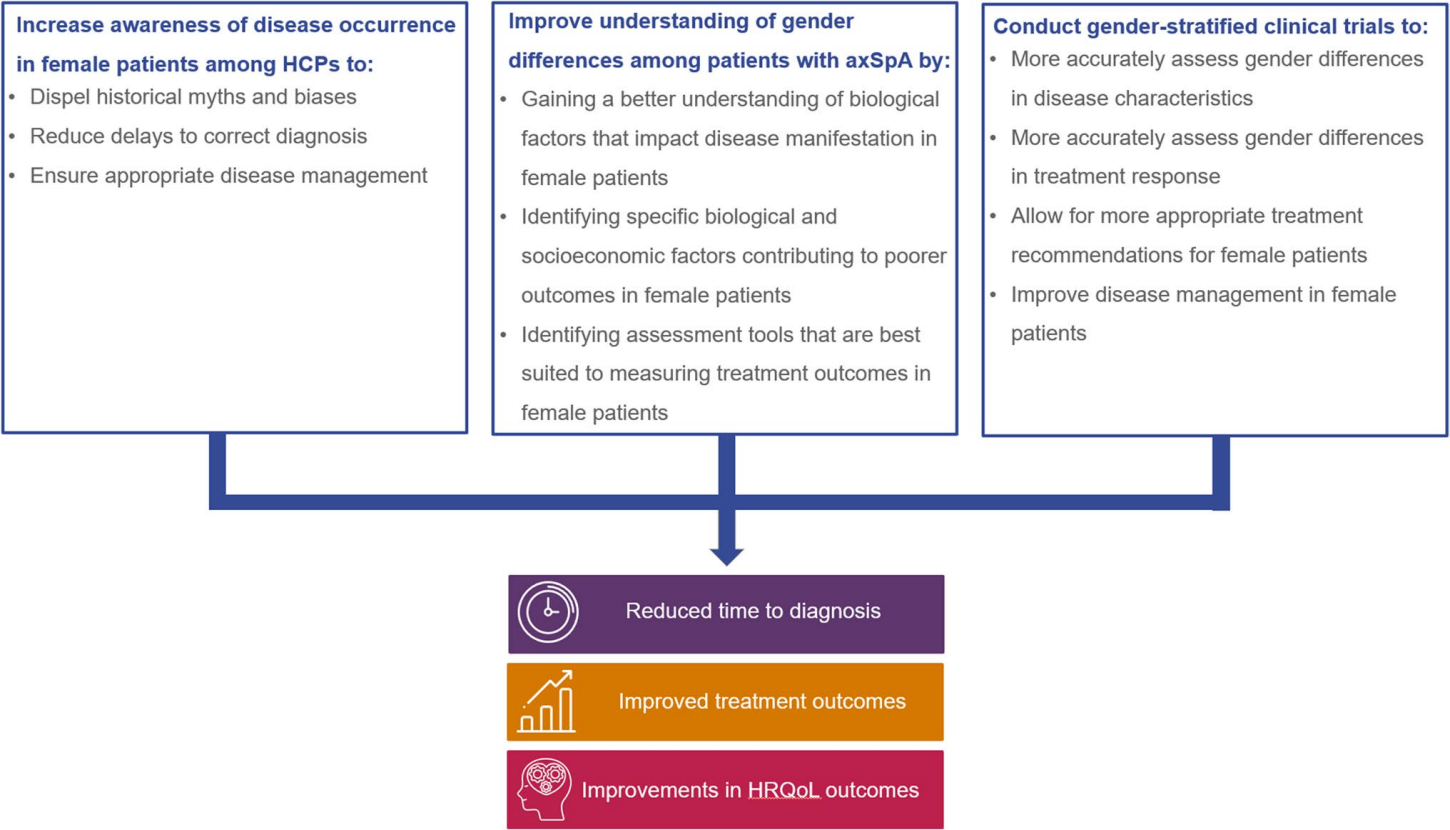
Three areas of education and research needed to address unmet needs in female patients with axSpA. Abbreviations: axSpA, axial spondyloarthritis; HCP, healthcare professional; HRQoL, health-related quality of life

To address the gender disparities and unmet needs in female patients with axSpA, we suggest three key areas of education and research that should be prioritised (Fig. 1). Firstly, there is a need to identify ways to further HCP training, particularly among primary care physicians, to raise awareness of gender differences in patients with axSpA. Education of HCPs plays a crucial role in reducing diagnostic delay-related disease burden. Improving education surrounding the signs, symptoms and management of axSpA is emphasised in EULAR recommendations and in many training initiatives including those from patient societies [19, 62]. However, further HCP training is required to dispel current myths surrounding axSpA and to raise awareness of the differences in disease manifestation and pain perception between men and women. Closer collaboration should also be encouraged between primary HCPs and rheumatologists and/or other related specialists (such as dermatologists and gastroenterologists), and improvements should be made to standardise the interpretation of MRI scans during the pathway to diagnosis [21]. More training would help HCPs correctly diagnose axSpA in a timely manner, which would be particularly beneficial to female patients [19].

Secondly, more research should be conducted to better understand biological differences between men and women, generally, and among patients with axSpA. Currently, research into new treatment options is accelerating at a faster rate than research into the biological sex differences between males and females with axSpA. Consequently, despite the availability of different treatment options, there is still a lack of evidence regarding the success of treatments for women with axSpA. A thorough exploration of the biological sex differences could help to explain the differences in disease manifestation and the factors contributing to poor treatment outcomes. This could also lead to improvements in disease assessment tools utilised in clinical trial settings. Currently, some outcome measures (e.g. BASDAI) incorporate assessments of pain and enthesitis, which are experienced differently by female patients with axSpA [46]. A better understanding of why women experience pain differently could lead to the introduction of gender-adjusted tools, which would allow a more accurate assessment of treatment outcomes in females, leading to more appropriate treatment strategies.

Thirdly, gender-stratified clinical trials should be conducted with an appropriate representative sample of female patients and designed to specifically examine gender differences in terms of disease manifestation and treatment response. The selection criteria for these trials should be adapted from current criteria, such as using positive MRI rather than a contextual assessment of inflammation. Such trials would allow for a thorough analysis of the gender differences in patient demographics and characteristics, which would build on the currently limited evidence base of gender differences in disease manifestation. Potential gender differences in terms of treatment adherence and response also need to be addressed, with an aim to identify more appropriate treatment strategies for women. This approach has been advocated for in other disease areas where there is a better understanding and awareness of gender differences, such as ischemic heart disease, also historically perceived as a male disease [63].

In conclusion, there exist gender differences in terms of time to diagnosis, treatment outcomes and HRQoL in patients with axSpA. Reflecting on the existing evidence, we propose three priority areas for change: the identification of ways to increase awareness of disease occurrence among HCPs, improve understanding of gender differences in disease manifestation and outcome measures, and conduct gender-stratified clinical trials. In our opinion, addressing these research needs would generate important evidence to help ensure timely diagnosis and appropriate disease management for women living with axSpA.

## Supplementary Information

Below is the link to the electronic supplementary material.

Supplementary file1 (DOCX 14.5 KB)

## Footnotes

1 We primarily use “gender differences” to acknowledge a person’s identity as a man or woman and their behaviours and attributes. The term “biological sex differences” is used only to refer to the differences in biological processes between males and females.
