# Supplementary material for: The impact of gender and sex on diagnosis, treatment outcomes and health-related quality of life in patients with axial spondyloarthritis
Source: Clin Rheumatol. 2022 Jun 28;41(11):3573–81. doi: 10.1007/s10067-022-06228-6 (PMC9568456; doi:10.1007/s10067-022-06228-6)
Supplement: Supplementary file 1 — Supplementary file1 (DOCX 14.5 KB) [file 10067_2022_6228_MOESM1_ESM.docx]

**The Impact of Gender on Diagnosis, Treatment Outcomes and Health-Related Quality of Life in Patients with Axial Spondyloarthritis**

Helena Marzo-Ortega,^1^ Victoria Navarro-Compán,^2^ Servet Akar,^3^ Uta Kiltz,^4^
Zoë Clark,^5^ Elena Nikiphorou^6,7^

*^1^NIHR Leeds Biomedical Research Centre, Leeds Teaching Hospitals Trust and Leeds Institute of Rheumatic and Musculoskeletal Medicine, University of Leeds, Leeds, UK; ^2^University Hospital La Paz, IdiPaz, Madrid, Spain; ^3^Department of Internal Medicine, Division of Rheumatology, Katip Çelebi University, İzmir, Turkey; ^4^Rheumazentrum Ruhrgebiet, Herne, and Ruhr-Universität Bochum, Bochum, Germany; ^5^Patient Author, Norwich, UK; ^6^Centre for Rheumatic Diseases, King’s College London, London, UK; ^7^Rheumatology Department, King’s College Hospital, London, UK*

**Corresponding Author:** Helena Marzo-Ortega: [H.Marzo-Ortega@leeds.ac.uk](mailto:H.Marzo-Ortega@leeds.ac.uk);
NIHR Leeds Biomedical Research Centre, Leeds Teaching Hospitals Trust and Leeds, Institute of Rheumatic and Musculoskeletal Medicine, University of Leeds, Leeds, UK

**Journal:** Clinical Rheumatology

**Search strategy for pragmatic literature review**

We performed a comprehensive literature search using PubMed to identify peer-reviewed articles published between 01 January 2000 and 27 May 2021. Search terms included “axial spondyloarthritis”, “axSpA”, “ankylosing spondylitis”, “sex”, “gender”, “male”, “female”, “woman” and “women”. Studies were included if they reported results on disease activity, diagnosis, treatment outcomes or HRQoL in female patients with axSpA. A second, more targeted search of conference proceedings from 2019 to 2021 was conducted to identify post-hoc analyses from clinical trials reporting results for patients with axSpA stratified by gender. Congresses included European Alliance of Associations for Rheumatology (EULAR) and the American College of Rheumatology (ACR).

A total of 489 records were retrieved in our original electronic database search. After title and abstract review, 59 records were selected for full-text review. Of these, 35 were considered relevant. Supplementary hand searches of congress abstracts and bibliographies of relevant articles identified additional publications of interest.
